# Investigation of sedimentary rocks and grains in the round: Micron‐scale SEM‐BEX 360° and 3D imaging

**DOI:** 10.1111/jmi.13389

**Published:** 2025-02-11

**Authors:** Jim Buckman

**Affiliations:** ^1^ Centre for Environmental Scanning Electron Microscopy (CESEM), Institute of GeoEnergy Engineering, School of Energy, Geoscience, Infrastructure and Society Heriot‐Watt University, Riccarton Edinburgh Scotland

**Keywords:** 360° panorama, large chamber SEM, Unity‐BEX detector, virtual reality (VR)

## Abstract

The aim of the current paper is to demonstrate the use of a novel prototype horizontal rotation stage, within a scanning electron microscope (SEM). Here we utilise sedimentary rock chips and grains, with images collected every 10 ° of rotation, to produce a continuous overlapping field of view 360° montage. The same procedure can also be used to collect elemental X‐ray maps. The collected images and maps can be simply combined into an image stack and saved as a movie format of the rotating specimen through 360°. More significantly, individual images and elemental maps can be reconstructed using photogrammetry imaging software, producing three‐dimensional (3D) models with mesh worked surfaces and rendered texture (colour or greyscale) based on SEM image and X‐ray data. This produces photorealistic smoothly transitioning movies with 360° fields of view around each object and can also be saved for stereo‐viewing using a virtual reality (VR) headset. This new technique generates highly detailed information on the 3D structural and compositional relationships between components at the submicron to millimetre scale. The technique has widespread applications, including imaging other geological materials, biological mineralised tests and material science specimens such as metals, ceramics and building materials.

## 1 INTRODUCTION

Imaging by scanning electron microscopy (SEM) typically produces 2D greyscale micrographs of relatively flat often irregularly shaped three‐dimensional sample materials. Samples can be rotated around a microscope's vertical axis, and in some cases tilted at up to 90° to view from different perspectives, and various methods exist to produce 3D images of such surfaces.^1^, ^2^, ^3^, ^4^, ^5^ Nevertheless, only the surface that faces the SEM pole piece can be imaged, making it difficult to view the surface of a sample in its entirety around 360° (i.e., top, bottom and all sides). In addition, it is possible to colourise SEM images to enhance interpretation and to utilise elemental X‐ray data.^6^ The latter provides additional information on the occurrence and distribution of different mineralogical phases, but again is limited in the scope of the surfaces that can be imaged and analysed by the same constraints as those for SEM image acquisition. Recently, it has also be shown how individual millimetre sized particles can be imaged using smartphone technology to create useful 3D models of geological grains.^7^

We have developed a simple stage modification for large‐chambered SEM's that allows for the conversion of rotation around the vertical axis into rotation around the horizontal axis. Under these conditions, it is possible to acquire images of small samples (less than 4 mm diameter) around a 360° horizontal axis, which can then be stitched together and rendered as realistic 3D objects using photogrammetry image processing software. This has potential for SEM‐X‐ray imaging in many fields of investigation, including the earth, materials, and biological sciences. Here we illustrate its use looking at the form and composition of sand grains and sandstone chips.

## 2 MATERIALS AND METHOD

This technique is applicable to a range of materials, but here is restricted to the examination of a mixed composition beach sand, Compton Bay, Isle of Wight, and two potential reservoir rock white sandstones (provenance unknown) presented as millimetre scale rock chips.

We used a Quanta FEG 650 scanning electron microscope (SEM), equipped with an Oxford Instruments Unity‐BEX detector for backscattered (BSE) imaging and qualitative elemental mapping and an X‐Max^N^ 150 mm energy dispersive X‐ray (EDX) detector. Also fitted with an in‐house prototype horizontal rotation stage (Figure 1), with samples superglued to a steel pin. The horizontal rotation stage attaches to the rotation stage, converting rotation around the vertical axis, through 90°, to produce rotation around a horizontal axis under the active SEM beam.

**FIGURE 1:**
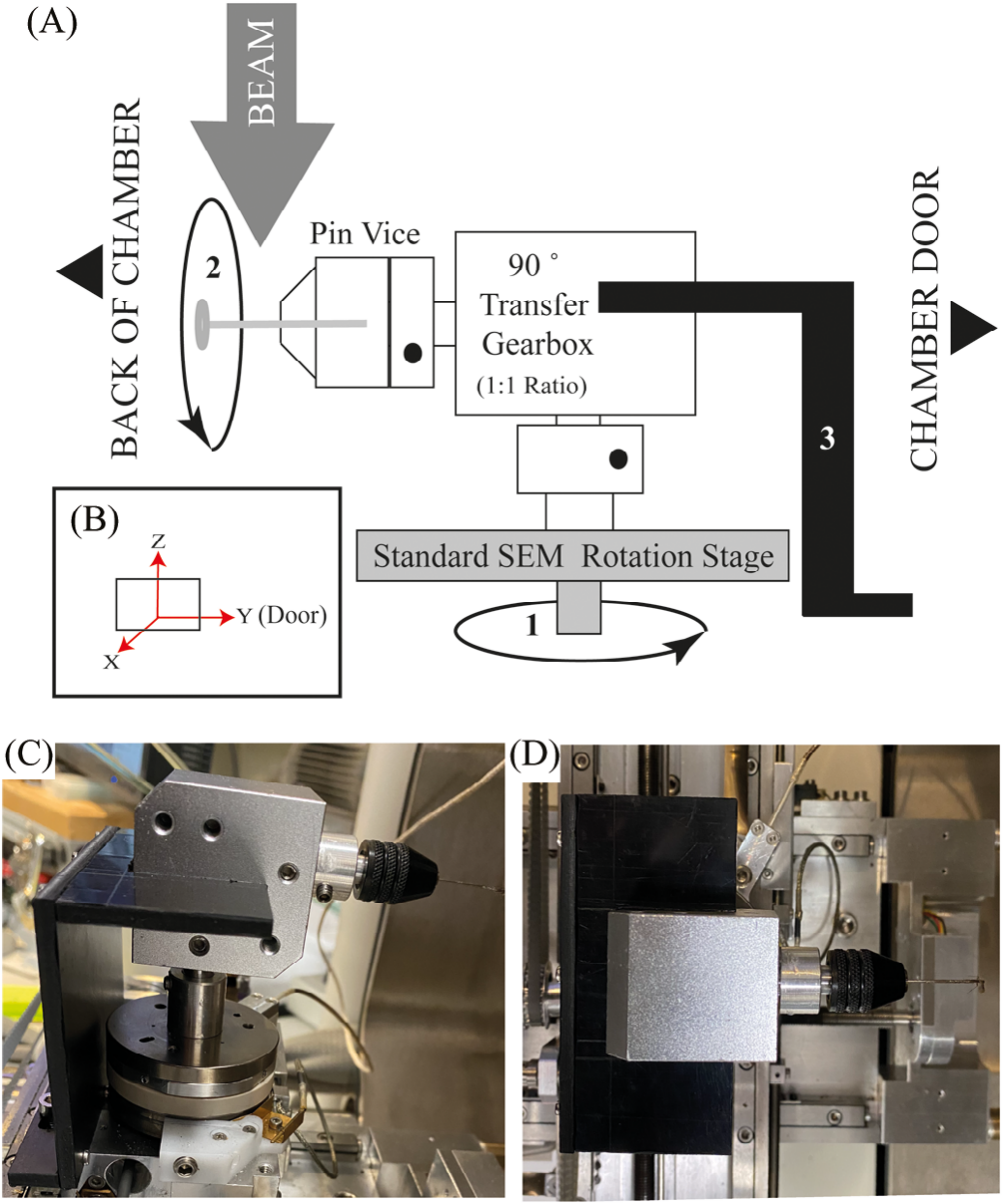
(A) Schematic vertical profile, through SEM chamber, of horizontal rotation stage, driven from preexisting standard SEM rotation stage. Note, rotation from standard SEM sample rotation stage (1), converted from rotation in the *X*–*Y* plane, to rotation in the X–*Z* plane (2) through a 90° transfer gearbox with a 1:1 ratio. (3) = retainer to prevent sample rotation in the *X*–*Y* plane. (B) Inset illustrating the plane of (A), relative to the SEM *X*–*Y*–*Z* axes. Images of horizontal rotation stage conversion, installed in Quanta 650 FEG SEM. (C) Side view, (D) top view.

Samples were held within the horizontal rotation stage using a built‐in pin vice, and the SEM pumped to low vacuum (0.83 Torr). A working distance of around 20 mm was used, and an operating current of 20 kV. Two methods were applied: (i) Imaging only, utilising the SEM built‐in Concentric Backscattered (CBS) detector. With a spot size of 4.5 and aperture selection of 6, used to maximise image quality and resolution. Images were typically collected with a scan time of 10 µs and with a horizontal pixel resolution of 1536 pixels. (ii) Combined BSE imaging and X‐ray mapping using an Oxford Instruments Unity‐BEX detector. Utilising a spot size of 5, and the widest available aperture setting (position 1), to maximise the X‐ray flux. In both cases, contrast and brightness where then adjusted to produce a suitable backscattered (BSE) image without too much contrast. In addition, scan‐rotation was set to 90° so that acquired images would rotate from left to right on the SEM screen. For BEX acquisition within the AZtec software we used a single 35 µs scan to simultaneously acquire both BSE image and X‐ray map, with a horizontal resolution of 1024 pixels. The magnification/horizontal field of view varied dependant on the size of the sample being scanned.

The sample was then centred on the SEM screen and dependant on the method being used, either CBS images or combined BSE X‐ray (BEX) images were taken every 10° of stage rotation. Due to mechanical backlash and difficulty in centrally mounting of some specimens, the image field of view was where necessary recentred for individual acquisitions. Contrast and brightness were not adjusted during the acquisition phase.

For BEX images, where elemental distribution maps were collected, the individual elements of interest were selected or deselected and represented with a unique colour value. Colours were chosen by trial and error, and selected to help separate mineral phases through the careful mixing of the colours for chosen elements.^8^

The collected images where initially imported into Agisoft Metashape standard V. 2.1.0 multiview 3D reconstruction software, which was used for post‐processing of the collected image/BEX images, following the protocol in Figure 2. Once processed the constructed models were saved as 360° rotating movies (.avi files), and in some cases were processed as stereo image pairs suitable for viewing with virtual reality (VR) headsets. As an alternative, images could be uploaded into FIJI/ImageJ as an image sequence and converted into an .avi movie. Such results are not as smooth in the nature of the .avi movies produced and typically require an additional level of care in acquiring individual frames, which typically need manual image recentring during acquisition. The latter is therefore not illustrated herein.

**FIGURE 2:**
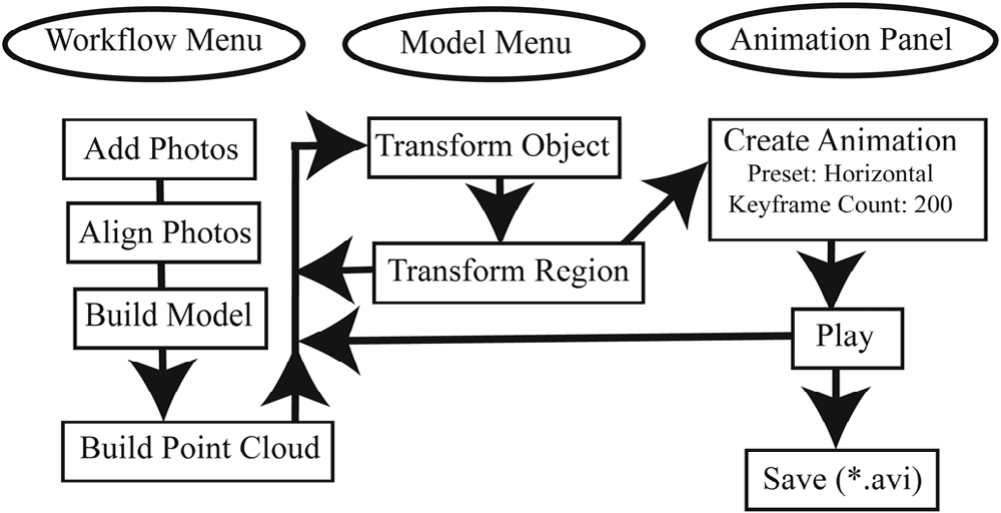
Workflow using Agisoft Metashape standard software, to construct 3D model from SEM images (or BEX image/X‐ray maps).

## 3 RESULTS

The technique was successfully applied to the 360° imaging of the surfaces of individual sand grains stuck to a steel pin (Figure 3), as well as to a small sandstone chip (Figure 4). In both cases the greyscale in the BSE images illustrates the occurrence of different mineral phases across the surface of the samples. Combined BSE and X‐ray (BEX) images were also successfully collected in the same manner and used to illustrate an example of sand grains on a steel pin (Figure 5), and compositional and grain variation in another sandstone chip (Figure 6). In addition, 3D rendered rotating images were successfully collected and tested for display using a VR headset (Figure 7).

**FIGURE 3:**
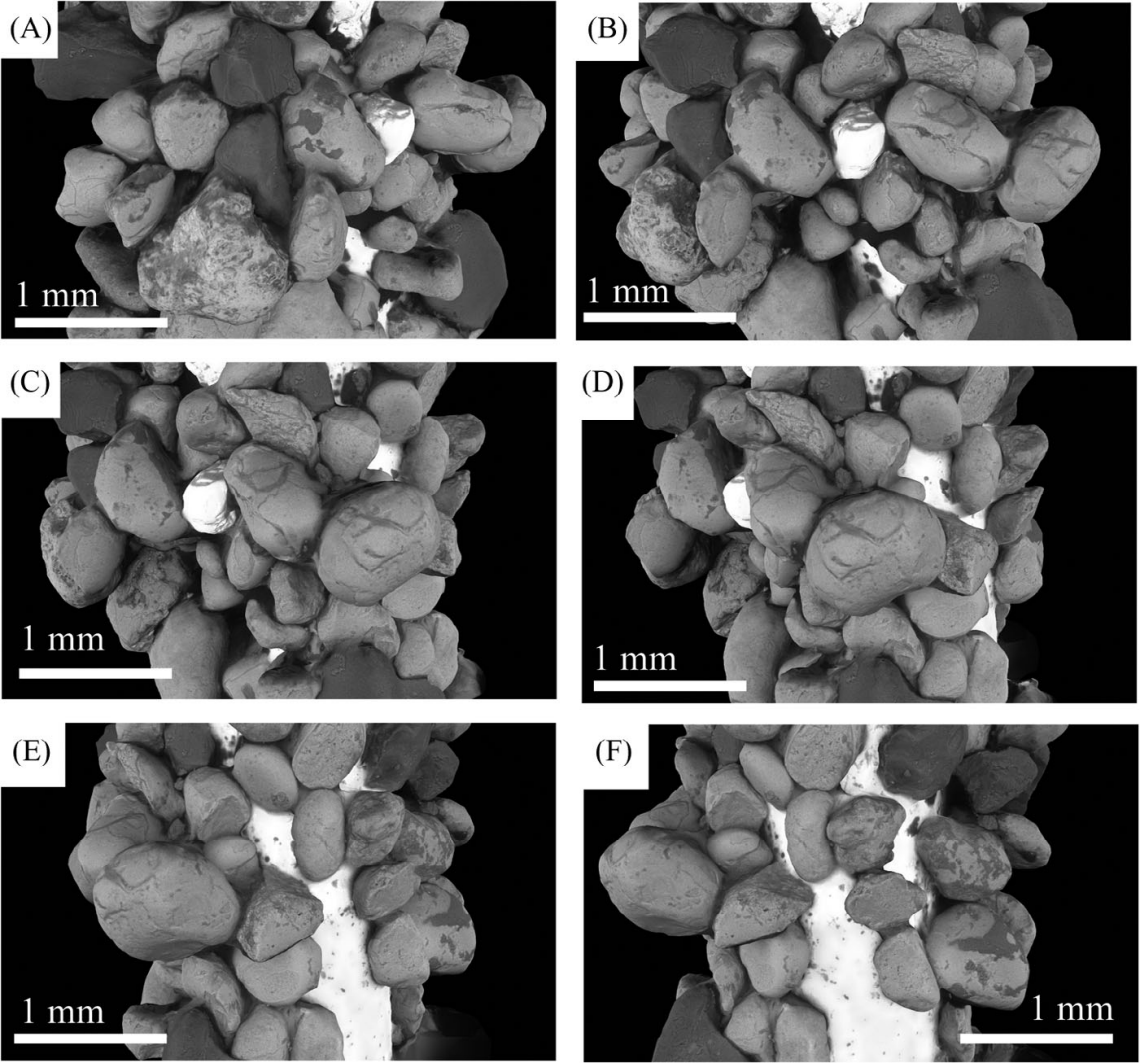
Six still images taken from 360° rotating backscattered (BSE) rendered video of sand grains glued to steel pin. See Supplementary Material for rotating .avi file.

**FIGURE 4:**
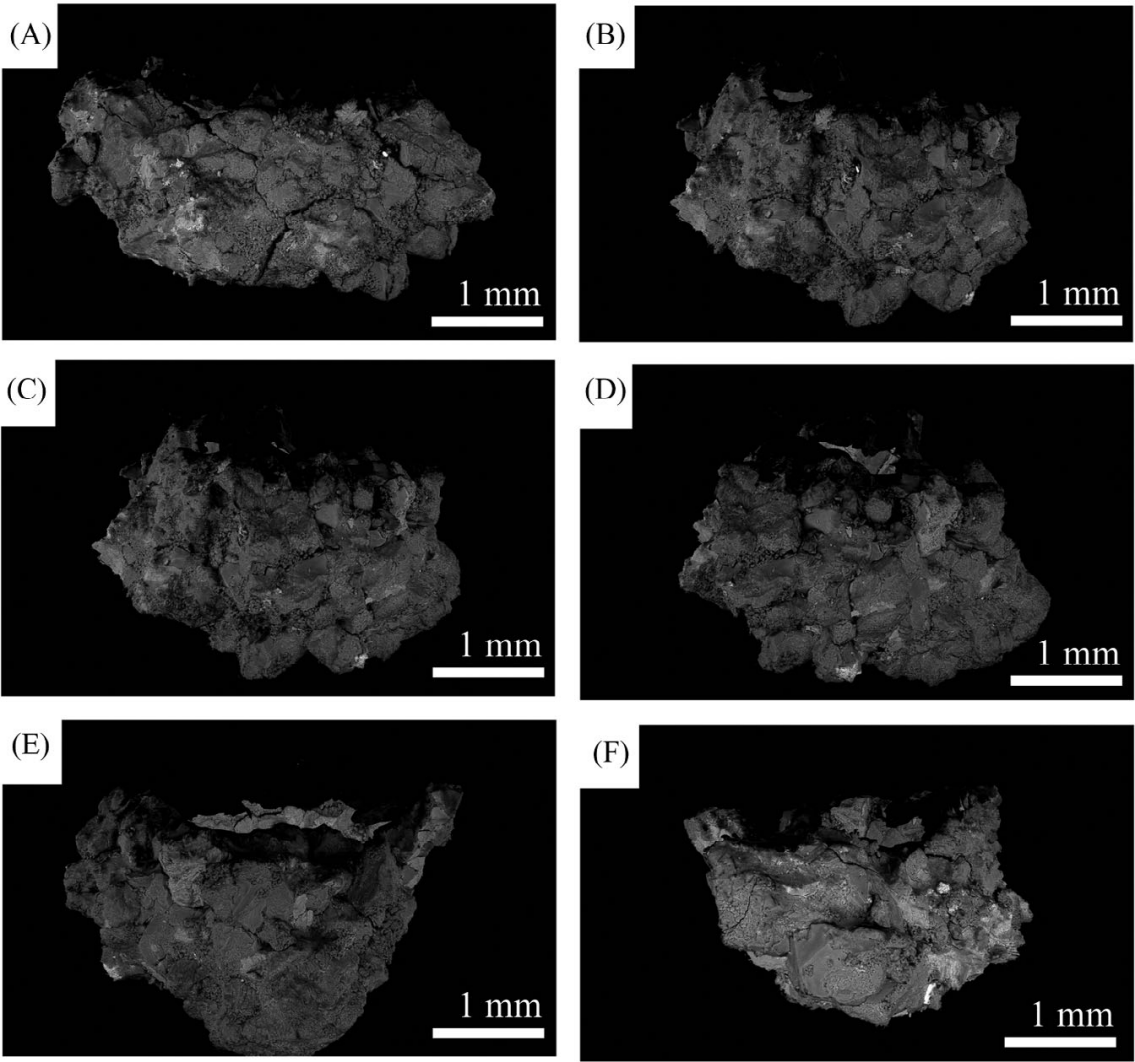
Six still images taken from 360° rotating backscattered (BSE) rendered video of sandstone chip. See Supplementary Material for rotating .avi file.

**FIGURE 5:**
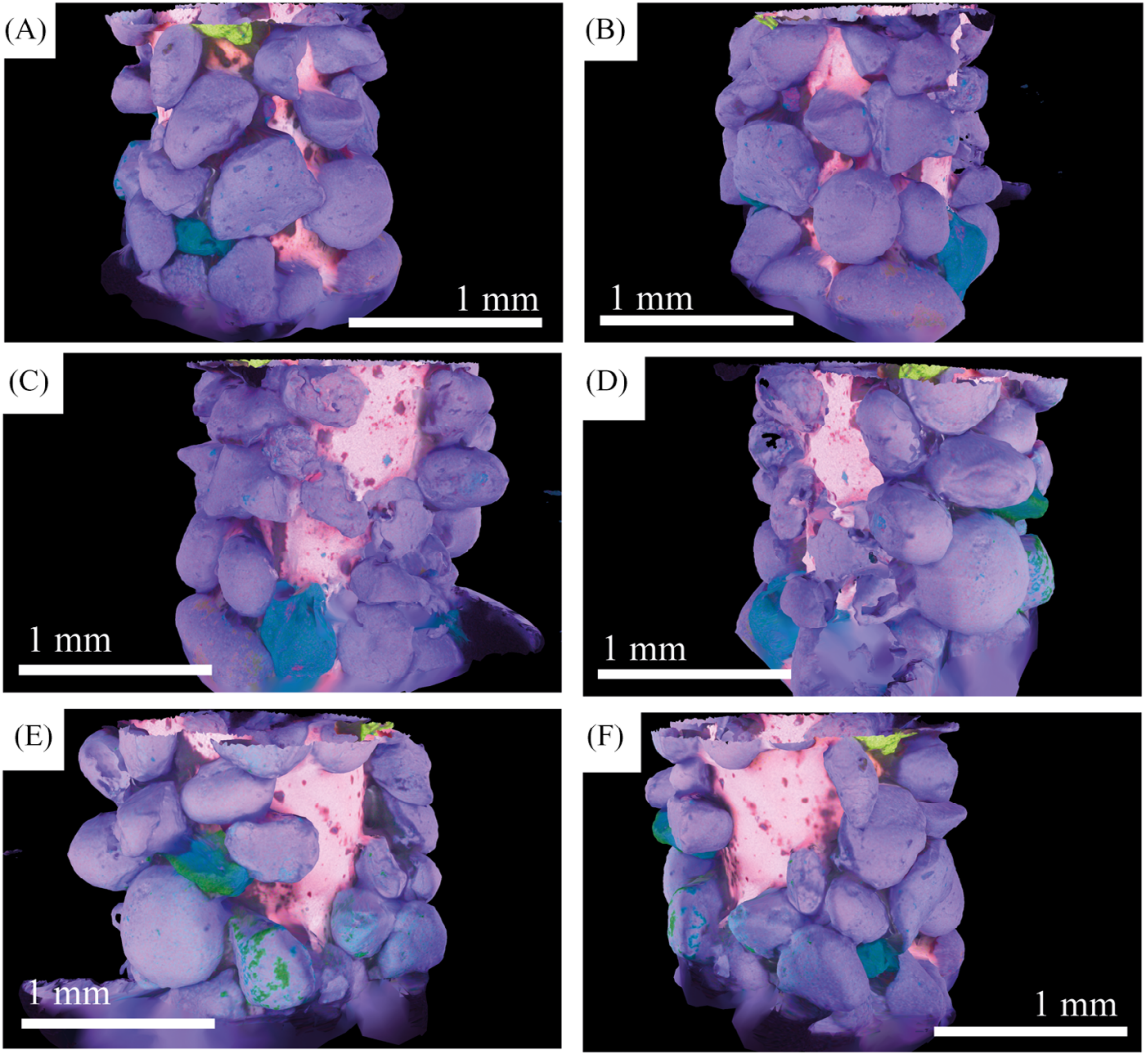
Six still images taken from 360° rotating BEX image rendered video of sand grains glued to steel pin. Purple = quartz, blue and green = feldspars or clays, pink = steel pin. See Supplementary Material for rotating .avi file.

**FIGURE 6:**
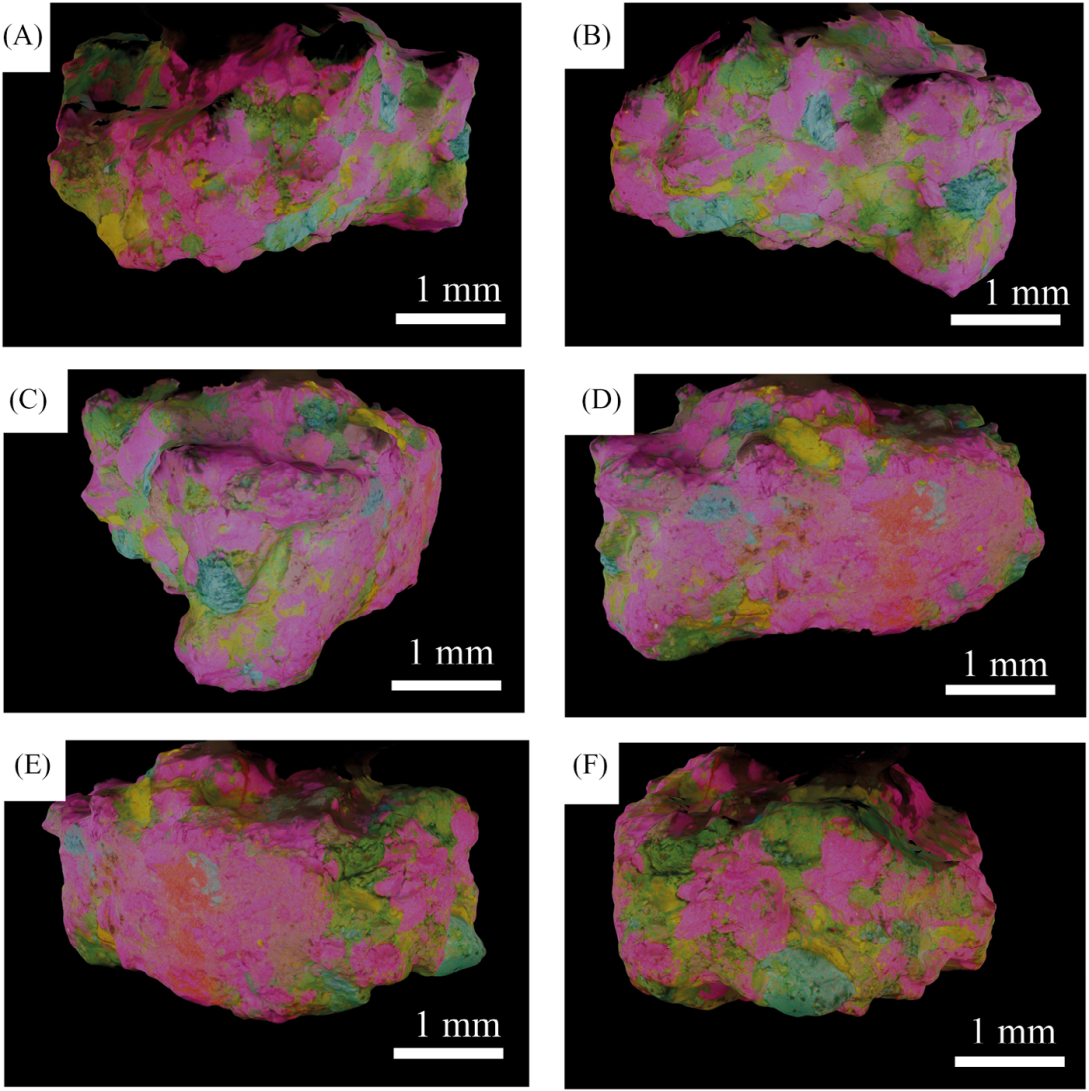
Six still images taken from 360° rotating BEX image rendered video of a second sandstone chip. Pink = quartz, teal and green = feldspars. See Supplementary Material for rotating .avi file.

**FIGURE 7:**
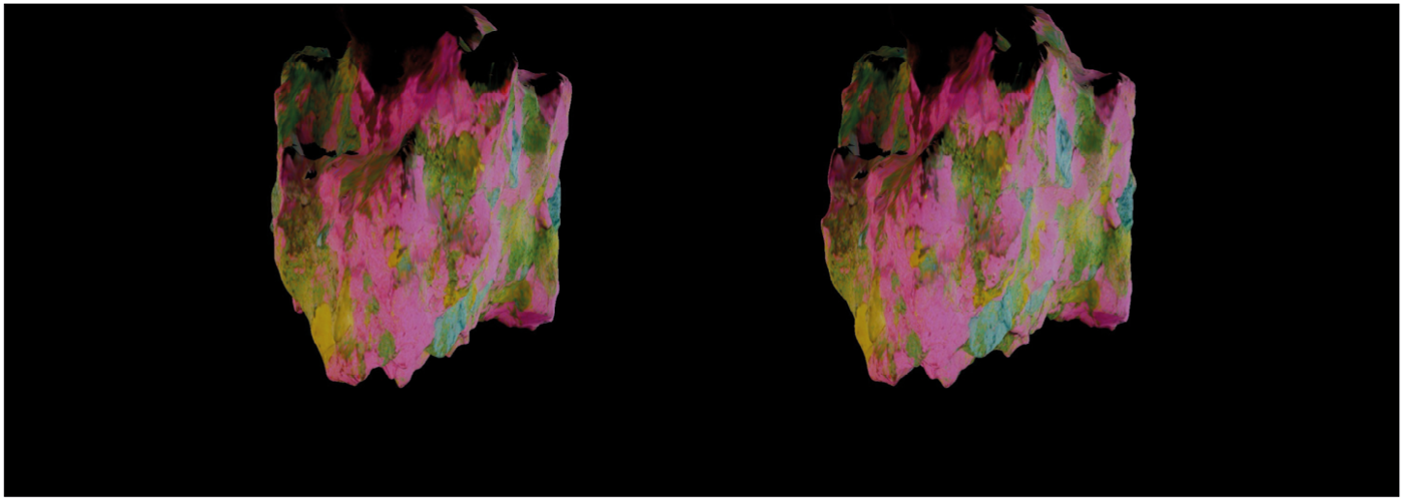
Stereo‐pair of rotating sandstone chip (as in Figure 6), for viewing with VR headset. PowerPoint file available in Supplementary Material.

## 4 DISCUSSION

The main reason for the construction of the stage was to test a means of obtaining high‐resolution SEM images of pores and grains within reservoir and seal rocks, which could be realistically modelled in 3D, including the generation of topographic surface meshworks (Figure 8). This successfully allows detailed imaging and analysis at the micron to submicron scale of structural and compositional elements, relating to grains, cements and pores in a 360° 3D format. Such information is of significance for a number of net zero carbon projects, for the reduction of greenhouse gas emissions, such as CO_2_ and H_2_ storage in reservoir rocks. In addition, this technique is not limited to the type of material illustrated in the current study. It was also possible to image the calcite tubes of the polychaete *Spirorbis* (Figure 9), and the technique clearly has applications in the imaging of other carbonate or silicate shell secreting microorganisms, such as foraminifera, diatoms, radiolaria and testate amoebae, which we are currently developing. Other areas such as the study of building materials (mortar, cement etc.), as well as research where it is desirable to see microscopic structural and elemental composition details in a 360° view/3D fashion will also likely benefit from this new technique.

**FIGURE 8:**
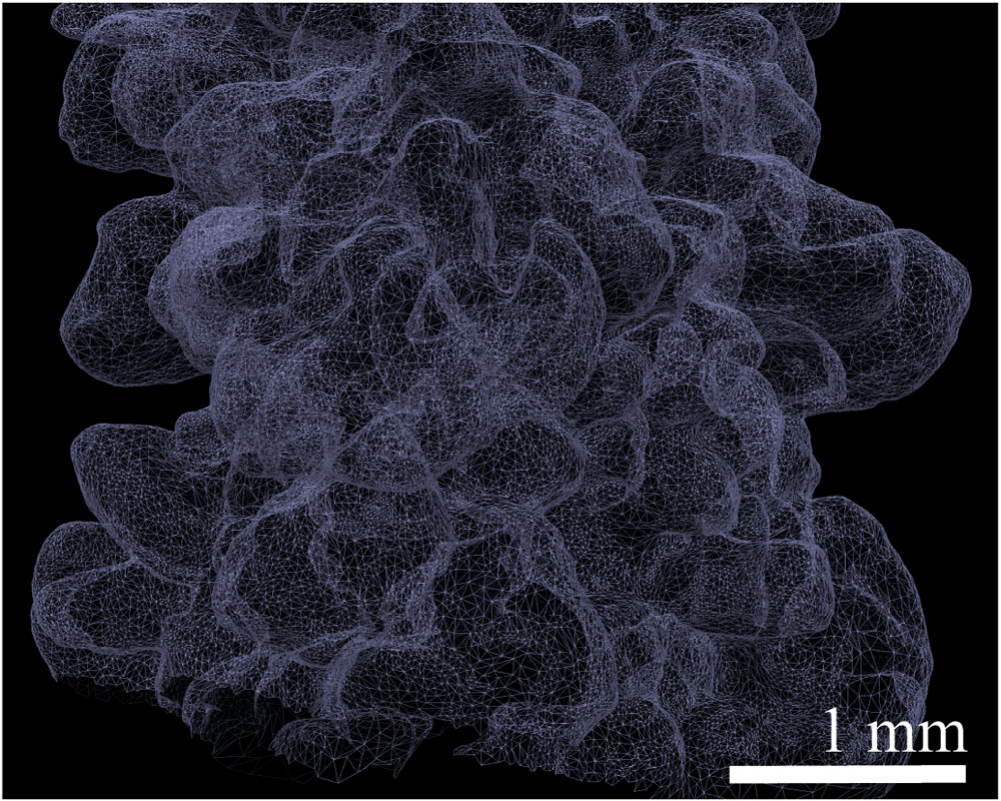
Still image taken from 360° rotating surface meshwork image of sand grains on steel pin. See Supplementary Material for rotating .avi file.

**FIGURE 9:**
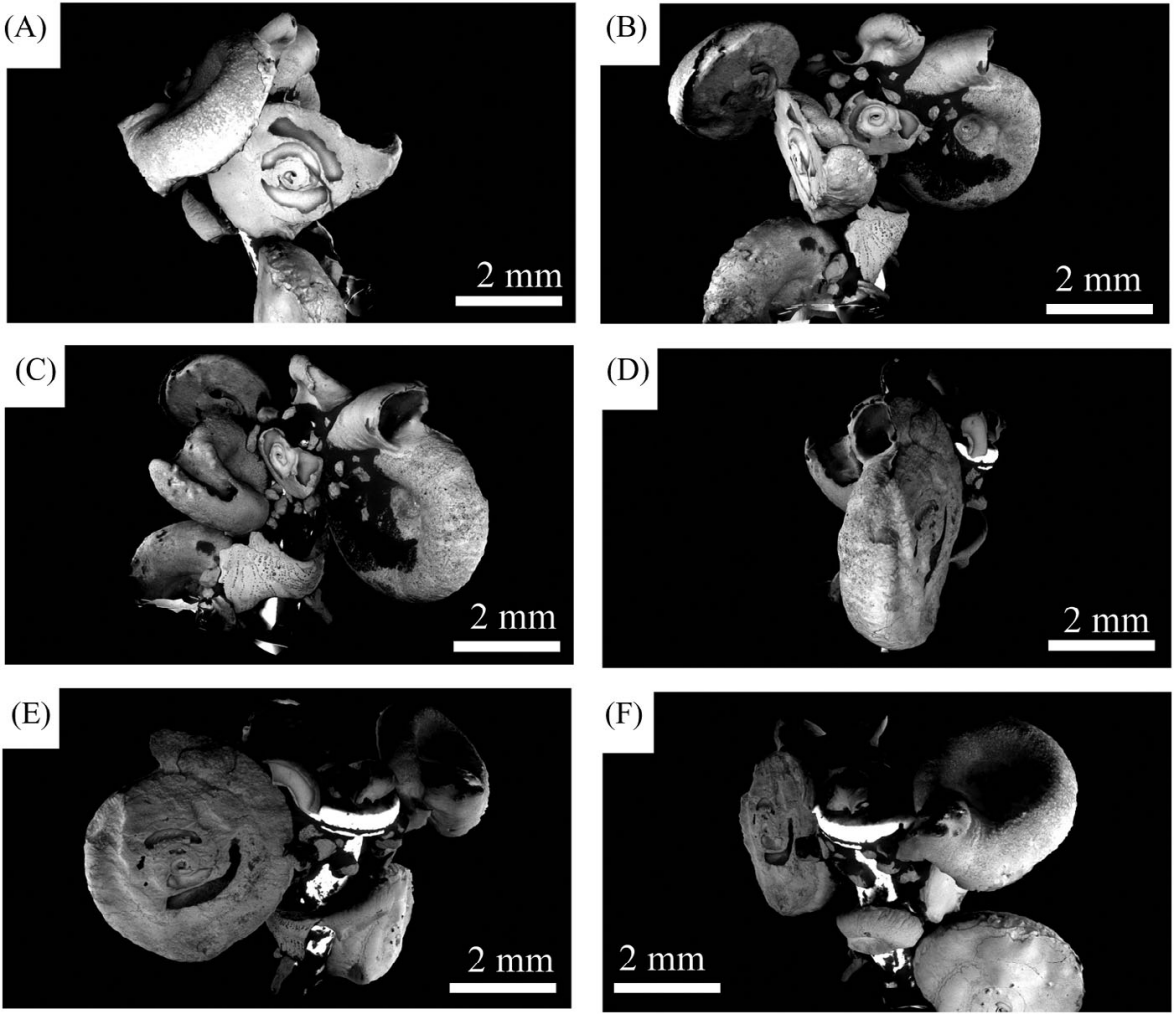
Six still images taken from 360° rotating backscattered (BSE) rendered video of the calcitic tube of the polychaete worm *Spirorbis*. See Supplementary Material for rotating .avi file.

This technique is not limited to the use of BSE images. It is equally possible to utilise secondary electron (SE), as well as potentially cathodoluminescence (CL) imaging, either on their own or in combination with fast elemental analysis (i.e., Unity‐BEX).

As the current software used can also generate 3D meshwork representations of the scanned samples, this allows for the exportation of acquired surfaces for further manipulation in other software packages, as well as the possibility of 3D printing at a larger scale for better visualisation in the real world.

Rendered results are similar to those obtainable with μ‐CT (X‐ray tomography) reconstruction of external surfaces (Figure 10). However, resolution is potentially higher and presents direct information on the chemical composition of mineralogical phases as well as their relative distribution and interrelationships. The degree of resolution obtained for each sample will ultimately depend on the horizontal field of view and pixel resolution used in the scanned images. In the current examples, this equates to around a maximum of 2 to 3 µm per pixel. However, this can easily be adjusted to a submicron resolution through use of smaller samples and/or the selection of a higher pixel density (i.e., 2048, 4096 pixel horizontal field of view per image).

**FIGURE 10:**
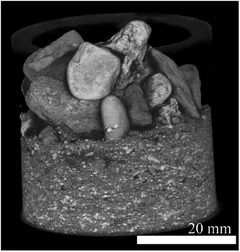
Still image of 360° rotating X‐ray tomography‐based reconstruction of the surface of sand and pebbles in a jar. See Supplementary Material for rotating .avi file.

The rendered images constructed using the current software are based on 3D meshworks created by the Metashape software, which is used to form a solid model on which texture is added based on the SEM /BEX images. In the majority of cases, this produces excellent photorealistic results. However, it should be noted that artefacts such as holes, and in some cases distorted parts of the reconstructed image may occur (see bottom of Figure 5D). In addition, isolated floating particles can occur on reconstructions, although where present such images can be reprocessed, or removed though the software.

## 5 FUTURE WORK

Currently we were only able to scan samples with a diameter of up to about 4 mm. This restriction is primarily due to the use of apertures within the final aperture and lens assembly. Where SEM systems are apertureless (e.g., Tescan VEGA and MIRA SEM), it should be possible to image larger centimetre sized objects using this same technique, and by using images with higher pixel resolutions still maintain details of micron sized structures.

The Agisoft Metashape reconstruction software has the option to utilise multiple sets of data taken from different vantage points around an object. Therefore, once a sample has been scanned within the SEM, it would be feasible to unmount the sample and remount at 90° to the original scan axis. This would result in two orbital views of a sample, which would be used to generate a textured model of the complete surface of the object. Once obtained, this would allow the possibility of generating a virtual 3D model, that could be viewed using virtual reality headsets and manipulated in any direction to view micron to submicron structural and compositional variation. Such would be an ideal tool for teaching and learning, and applicable to a range of subjects. This could be developed for the study of foraminifera and individual sand grain particles. A new stage is currently under development that should allow such new work.

Future work currently under development will facilitate the imaging of surfaces from BEX scans obtained from small core plugs, allowing comparison and correlation with μ‐CT 3D analysis of the same core plugs. This would allow the integration of higher‐resolution external surface details with typically lower resolution μ‐CT reconstructions, to enhance and better resolve internal structure (i.e., pore, grain and cement form and geometry). As both μ‐CT reconstructed slices and BSE images vary in their greyscale level based on molecular density, the BEX elemental data could be used to directly map on to the μ‐CT scanned volume and produce coloured core scans based on composition and identify the mineral phases present. The latter would greatly enhance the interpretation of such scans and be applicable to many areas where knowledge of porosity, pore throat connectivity, development of cement, the occurrence of dissolution features, and relative distribution of mineral phases is of interest (such as CO_2_ and H_2_ storage, geothermal energy projects, oil and gas exploration, cement technology, mortar carbonation etc.; see Ref. [9]). As such, this may have a significant role in a range of net zero carbon projects.
